# Supplementary material for: Cumulative burden of non-communicable diseases predicts COVID hospitalization among people with HIV: A one-year retrospective cohort study
Source: PLoS One. 2021 Dec 1;16(12):e0260251. doi: 10.1371/journal.pone.0260251 (PMC8635326; doi:10.1371/journal.pone.0260251)
Supplement: S1 Table — (PDF) [file pone.0260251.s001.pdf]

Table 1. Demographics and clinical characteristics of PWH with SARS-CoV-2, 1/21/20-1/20/21 (n=103)						
	**Total (n=103)**	**Ambulatory (n=69)**	**Hospitalized (n=34)**	**p-value**	**OR (95% CI)**	**aOR (95%CI)[&]**
**Demographics**						
Median age (IQR)	56 (45 - 62)	55 (42.5-59)	58 (52.3-67.3)	0.40[#]		
Age≥50yrs n(%)	72 (69.9)	45 (65.2)	27 (79.4)	0.14		
Age>65yrs n(%)	18 (17.5)	8 (11.6)	10 (29.4)	**0.025**	**3.18 (1.12-9.01)**	3.11 (0.97-9.98)
Women n(%)	48 (46.6)	32 (46.4)	16 (47.1)	0.95		
African Americans n(%)	45 (43.7)	30 (43.5)	15 (44.1)	0.95		
Latinx n(%)	17 (16.5)	13 (18.8)	4 (11.8)	0.27		
**HIV History**						
Median years living with HIV (IQR)	16.5 (9 – 23.8)	17 (8-25)	16 (10-23)	0.83[#]		
History of AIDS n(%)	46 (44.7)	30 (43.5)	16 (47.1)	0.75		
History of CD4<200 cells/mm3 n(%)	43 (41.8)	28 (40.5)	15 (44.1)	0.77		
History of OI n(%)	24 (23.3)	18 (26.1)	6 (17.6)	0.25		
On ART n(%)	101 (98.1)	67 (97.1)	34 (100)	0.32		
Median CD4 count (IQR)	735 (434-928)	718 (397-931)	784 (466-924)	0.67[#]		
Proportion VL suppressed (VL<200) n(%)	95 (92.2)	65 (95.2)	30 (88.2)	0.25		
**Comorbidities**						
Active smoking n(%)	23 (22.3)	15 (21.7)	8 (23.5)	0.84		
Former smoking n(%)	58 (56.3)	37 (53.6)	21 (61.8)	0.43		
Active substance use n(%)	14 (13.6)	6 (8.7)	8 (23.5)	**0.039**	**3.2 (1.0-10.2)**	
Active alcohol use n(%)	28 (27.2)	19 (27.5)	9 (26.5)	0.91		
Diabetes Mellitus n(%)	28 (27.2)	14 (20.3)	14 (41.2)	**0.025**	**2.75 (1.1-6.8)**	

Median HgbA1C (IQR) (n=50)	7.8 (6.4-8.5)	7.1 (6.2-8.3)	8.3 (6.6-9.0)	0.20#		
Chronic Lung Disease n(%)	35 (34)	18 (26.1)	17 (50)	**0.016**	**2.83 (1.20-6.7)**	**3.35 (1.28-8.72)**
Chronic Kidney Disease n(%)	23 (22.3)	10 (14.5)	13 (38.2)	**0.007**	**3.65 (1.39-9.57)**	
Cardiovascular Disease n(%)	28 (27.2)	13 (18.8)	15 (44.1)	**0.007**	**3.4 (1.37-8.42)**	**3.4 (1.27-9.12)**
Hypertension n(%)	53 (51.5)	31 (44.9)	22 (64.7)	0.059	2.25 (0.96-5.25)	
Hepatitis C n(%)	23 (22.3)	11 (15.9)	12 (35.3)	**0.027**	**2.88 (1.1-7.47)**	
Median BMI (IQR)	28.9 (25.2-34.9)	29.3 (25.7-35.0)	26.8 (24.7-36.0)	0.30#		
Obesity (BMI>30) n(%)	45 (43.7)	32 (46.4)	13 (38.2)	0.43		
Any comorbidity n(%)	93 (90.3)	62 (89.9)	31 (91.2)	0.83		
Number of Comorbidities	3 (1-4)	2 (1-3)	4 (2-5)	**0.001&**	**1.61 (1.22-2.13)**	

#Mann Whitney U; &logistic regression; PWH-People with HIV; BMI-Body mass index
