# Supplementary material for: Cumulative burden of non-communicable diseases predicts COVID hospitalization among people with HIV: A one-year retrospective cohort study
Source: PLoS One. 2021 Dec 1;16(12):e0260251. doi: 10.1371/journal.pone.0260251 (PMC8635326; doi:10.1371/journal.pone.0260251)
Supplement: S2 Table — (PDF) [file pone.0260251.s002.pdf]

**Table 2. Characteristics of hospitalized PWH (n=34)**	
**Clinical parameters**	
Median Temperature on admission, degrees C (IQR)	37.5 (36.8 - 38.2)
Median CD4 Count cells/mm3 during hospitalization (n=15)	417.8 (186 – 617)
HIV Viral Suppression during hospitalization (<200 copies/mm3) (n=20)	19 (95%)
**COVID therapy**	
Hydroxychloroquine	16 (47.1%)
Tocilizumab	8 (23.5%)
Remdesivir	5 (14.7%)
Methylprednisolone	5 (14.7%)
Atazanavir	3 (8.8%)
Lopinavir/Ritonavir	2 (5.9%)
Convalescent Plasma	0
No therapy	6 (17.6%)
**Clinical Outcomes**	
Length of Stay (days)	9 (3-15)
Escalation of care to critical care unit<br>Stepdown:<br>ICU:	11(32.4%)<br>6 (17.6%)<br>5 (14.7%)
Mechanical Ventilation	4 (11.8%)
Mortality (of hospitalized/of all PWH)	1 (2.94%/0.97%)
